# Elderly patients with fluoropyrimidine and thymidylate synthase inhibitor-resistant advanced colorectal cancer derive similar benefit without excessive toxicity when treated with irinotecan monotherapy

**DOI:** 10.1038/sj.bjc.6602169

**Published:** 2004-09-14

**Authors:** I Chau, A R Norman, D Cunningham, J S Waters, C Topham, G Middleton, M Hill, P J Ross, R Katopodis, G Stewart, J R Oates

**Affiliations:** 1 Royal Marsden Hospital, London and Surrey, UK; 2 St Luke's Oncology Centre, Guildford, UK; 3 Kent Oncology Centre, Maidstone, UK

**Keywords:** irinotecan, colorectal cancer, elderly, performance status, toxicity, survival

## Abstract

Elderly patients are recommended to have a reduced starting dose (300 mg m^−2^ once every 3 weeks) of irinotecan monotherapy. The aims of this analysis are to compare toxicity and survival according to age, performance status (PS), gender and prior radical pelvic radiotherapy (RT). The primary end points were overall survival and an irinotecan-specific toxicity composite end point (TCE) defined as the occurrence of grade 3 or 4 diarrhoea, neutropenia, febrile neutropenia, fever, infection or nausea and vomiting. Between 1997 and 2003, 339 eligible patients with advanced colorectal cancer (CRC) progressing on or within 24 weeks of completing fluoropyrimidine-based chemotherapy were prospectively registered in a multicentre randomised trial. All patients commenced irinotecan at 350 mg m^−2^ once every 3 weeks. There were no differences in proportions of patients developing TCE by age (<70 *vs* ⩾70 : 37.8 *vs* 45.8%; *P*=0.218), PS (0–1 *vs* 2 : 39.3 *vs* 41.5%; *P*=0.793) or prior RT (RT *vs* no RT : 45.1 *vs* 38.5%; *P*=0.377). Males experienced more toxicity than females (44.3 *vs* 32.6%; *P*=0.031), but this was not significant after controlling for other co-variates (*P*=0.06). Patients aged ⩾70 had similar objective responses (11.1 *vs* 9%; *P*=0.585) and survival (median 9.4 *vs* 9 months; log rank *P*=0.74) compared to younger patients. Elderly patients derive the same benefit without experiencing more toxicity with second-line irinotecan treatment for advanced CRC. Our data do not support the recommendation to reduce the starting dose for the elderly patients.

Elderly patients with colorectal cancer (CRC) have the same benefit from fluorouracil-based chemotherapy as younger patients in both adjuvant (Sargent *et al*, 2001; Sundararajan *et al*, 2002) and advanced disease (Popescu *et al*, 1999) settings. The role of irinotecan is now established in patients with fluoropyrimidine-refractory CRC based on two randomised studies demonstrating survival benefit over best supportive care or alternative schedules of infused fluorouracil (Cunningham *et al*, 1998; Rougier *et al*, 1998). Despite its efficacy, irinotecan produces toxicities that could be potentially life-threatening, especially when given with bolus 5-FU/leucovorin (LV) (Rothenberg *et al*, 2001). It is currently unclear whether elderly patients tolerate irinotecan poorly and whether a reduced starting dose for these patients is preferable. Moreover, the potential risks need to be weighed against the expected benefits of receiving irinotecan, especially in this older age group.

The dose adjustment guideline for irinotecan monotherapy, produced by the manufacturer which is in general circulation with clinicians, recommends a reduced starting dose of 300 mg m^−2^ once every 3 weeks for patients aged >70 years with World Health Organisation (WHO) performance status (PS) 2. This guideline is based on the two pivotal second-line studies – both recommended 300 mg m^−2^ for patients aged ⩾70 or PS 2 as these factors were previously recognised risk factors for developing toxicity (Cunningham *et al*, 1998; Rougier *et al*, 1998). One further phase III study comparing two irinotecan dose regimens in second-line therapy of metastatic CRC also made the same recommendation (Fuchs *et al*, 2003). However, none of these studies reported any data that prompted this recommendation of a reduced starting dose (300 mg m^−2^) for these particular patient populations. In addition, it is generally accepted that patients who had previous radical pelvic radiotherapy (RT) are also at risk of developing severe toxicity with irinotecan, especially diarrhoea. The aims of our analysis are to compare toxicity and survival according to age, PS, gender and prior radical pelvic RT in a group of patients treated within a prospective randomised controlled trial, all of whom commenced irinotecan at a dose of 350 mg m^−2^ given every 3 weeks.

## PATIENTS AND METHODS

We performed a phase III multicentre prospective randomised controlled trial recruiting patients from six oncology centres in the United Kingdom. The primary efficacy end point of this study has been reported previously (Lal *et al*, 2004). The eligibility criteria included: locally advanced or metastatic histologically proven colorectal cancer that progressed on or within 24 weeks of fluorouracil, raltitrexed or oral fluoropyrimidine-based chemotherapy; WHO PS ⩽2; bidimensionally measurable disease assessed by chest X-ray or computed tomography (CT) and satisfactory haematological, renal and liver functions. Patients who had received previous adjuvant chemotherapy and up to a maximum of three lines of palliative chemotherapy as well as those with no measurable disease were permitted into the study.

All patients fulfilling the eligibility criteria were prospectively registered for the trial. Patients who achieved a radiological objective response or disease stabilisation after 24 weeks of irinotecan were then randomly assigned to either stop irinotecan or continue irinotecan on a 1 : 1 basis using random permuted blocks. Patients were stratified according to number of previous lines of treatment. The protocol was approved by the Scientific and Research Ethics Committee of the participating institutions as well as the London Multicentre Research Ethics Committee. Written informed consent was obtained from each patient at registration.

Patients were treated with irinotecan 350 mg m^−2^ intravenously over 30 min every 3 weeks for eight cycles. No reduced starting dose was recommended in the protocol for patients aged ⩾70 years, WHO PS 2 or previous radical pelvic RT. Toxicity was measured using National Cancer Institute-Common Toxicity Criteria version 2. Radiological assessment with CT scan was made after every four cycles of irinotecan. Radiological tumour response was evaluated according to WHO Criteria (Miller *et al*, 1981). Complete response (CR) was defined as the complete disappearance of all measurable lesions, without the appearance of new lesion(s). Partial response (PR) was defined as a reduction of bi-dimensional lesions by ⩾50% of the sum of the products of the largest perpendicular diameters of each measurable lesion and no progression in other lesions or the appearance of any new lesions. Stable disease (SD) was defined as a <50% reduction of tumour volume or a <25% increase of the volume of one or more measurable lesions, with no new lesions. Progressive disease (PD) was defined as an increase of ⩾25% of the size of at least one bi-dimensionally measurable lesion, the appearance of new lesion(s), and/or the onset of ascites or pleural effusion with cytological confirmation.

## STATISTICAL CONSIDERATIONS

In this secondary analysis of the trial, the primary objectives were to compare toxicity and survival in the whole cohort of registered patients with the following subgroups which were set *a priori*: (i) aged <70 *vs* ⩾70 years; (ii) PS 0–1 *vs* 2; (iii) male *vs* female and (iv) previous pelvic RT (⩾45 Gy total dose) *vs* no pelvic RT. The primary end points were irinotecan-specific toxicity composite end point (TCE) and overall survival (OS). TCE was defined as the occurrence of either grade 3 or 4 diarrhoea, neutropenia, febrile neutropenia, fever, infection or nausea and vomiting. These toxicities were components of the gastrointestinal syndrome that caused early 60-day mortality related to irinotecan therapy (Rothenberg *et al*, 2001). Logistic regression modelling was used to compare different groups in the frequency of developing TCE. Overall survival was calculated from the date of registration until death from any cause or censored at last follow-up. Both time to developing first TCE and OS were calculated using the Kaplan–Meier method (Kaplan and Meier, 1958) and were compared between the groups using the log-rank test (Peto and Peto, 1972). Univariate analysis was performed using logistic regression and the log-rank test to identify characteristics predictive for occurrence of TCE and survival, respectively. The predictive factors analysed for effect were age (<70 *vs* ⩾70), PS (0–1 *vs* 2), gender (male *vs* female), previous pelvic RT (yes *vs* no), number of metastatic sites (0 or 1 *vs* >1), baseline serum alkaline phosphatase (⩽upper limit of normal range (ULN) *vs* >ULN), bilirubin (as a continuous variable as few patients had elevated bilirubin level due to trial eligibility), haemoglobin (⩽11 g/dl *vs* >11g dl^−1^) and white blood cell (⩽11 × 10^9^ l *vs* >11 × 10^9^ l^−1^) levels. Apart from the *a priori* defined comparison groups, other factors were chosen because of their prognostic value in patients treated with 5-FU based chemotherapy for metastatic CRC (Kohne *et al*, 2002). Multivariate survival analysis was performed using Cox's proportional hazards model (Cox, 1972) and corrected for all the significant prognostic factors. All end points were updated in December 2003. Analyses were performed using SPSS package version 12 (SPSS Inc., Chicago, IL, USA) and two-sided *P*-values <0.05 were considered statistically significant.

## RESULTS

Between November 1997 and May 2003, 348 patients were prospectively registered into this study. A total of 55 patients with responding or SD after eight cycles of irinotecan were randomised to stop irinotecan (*n*=30) or continue until disease progression (*n*=25). The efficacy of the randomisation part of the study is the subject of a separate publication. In this current analysis, nine (2.6%) patients were excluded due to reduced starting dose (300 mg m^−2^, *n*=5), ineligible patient (*n*=1), death before starting treatment (*n*=1) and no clinical data (*n*=2); therefore, 339 patients treated with irinotecan 350 mg m^−2^ once every 3 weeks were analysed.

Table 1

**Table 1:** Baseline characteristics at registration for the whole group

**Characteristics**	**Number of patients**
Total number of patients	339
Median age (years)	62
Range	29–80
<70 years old	267 (78.8%)
⩾70 years old	72 (21.2%)
	
*Gender*	
Male	201 (59.3%)
Female	138 (40.7%)
	
*Performance status*	
0	90 (26.6%)
1	205 (60.5%)
2	40 (11.8%)
3	1 (0.3%)
Unknown	3 (0.9%)
	
*Primary tumour sites*	
Colon	209 (61.7%)
Rectum	88 (26%)
Rectosigmoid junction	20 (5.9%)
Synchrounous	13 (3.8%)
Others	4 (1.2%)
Unknown	5 (1.5%)
	
*Involved disease sites*	
Liver	246 (72.6%)
Lung	143 (42.2%)
Peritoneum	55 (16.2%)
Primary tumour *in situ* or local recurrence	107 (31.6%)
	
*Previous radical pelvic radiotherapy*	
Yes	51 (15%)
No	288 (85%)

shows the baseline characteristics at registration for the whole group. The median age for patients in the <70 years age group was 58 (range=29–69) whereas the median age for those in the ⩾70 years age group was 72 (range=70–80). Only one patient had PS 3. Table 2

**Table 2:** Incidences of grade 3 or 4 toxicities

**Toxicities**	**Whole group (*n*=339)**	**<70 years (*n*=267)**	**⩾70 years (*n*=72)**	**Male (*n*=201)**	**Female (*n*=138)**	**PS 0–1 (*n*=295)**	**PS 2–3 (*n*=41)**	**Previous RT (*n*=51)**	**No previous RT (*n*=288)**
Anaemia	17 (5%)	15 (6%)	2 (3%)	10 (5%)	7 (5%)	16 (5%)	1 (2%)	3 (6%)	14 (5%)
Neutropenia	83 (24%)	58 (22%)	25 (35%)	61 (30%)	22 (16%)	72 (24%)	11 (27%)	12 (24%)	71 (25%)
Thrombocytopenia	12 (4%)	8 (3%)	4 (6%)	7 (3%)	5 (4%)	10 (3%)	2 (5%)	3 (6%)	9 (3%)
Febrile neutropenia	4 (1%)	2 (0.7%)	2 (3%)	3 (1%)	1 (0.7%)	3 (1%)	1 (2%)	0 (0%)	4 (1%)
Diarrhoea	53 (16%)	42 (16%)	11 (15%)	35 (17%)	18 (13%)	43 (15%)	9^a^ (22%)	12 (24%)	41 (14%)
Nausea and vomiting	18 (5%)	15 (6%)	3 (4%)	10 (5%)	8 (6%)	17 (6%)	1 (2%)	2 (4%)	16 (6%)
Infection	20 (6%)	18 (7%)	2 (3%)	14 (7%)	6 (4%)	17 (6%)	3 (7%)	4 (8%)	16 (6%)
Fever	13 (4%)	12 (5%)	1 (1%)	9 (5%)	4 (3%)	11 (4%)	2 (5%)	1 (2%)	12 (4%)
Lethargy	72 (21%)	57 (21%)	15 (21%)	45 (22%)	27 (20%)	60 (20%)	11 (27%)	10 (20%)	62 (22%)

PS: performance status. RT: radiotherapy.

a One patient with grade 3–4 diarrhoea had unknown baseline performance status.

shows the incidences of maximum grade adverse events occurring during any cycle in the whole group. Although the elderly had a higher incidence of neutropenia (*P*=0.0228), the incidences of infection, fever and febrile neutropenia were not significantly increased. Patients who had prior RT did not have a significantly higher incidence of diarrhoea compared to those who did not receive prior RT (*P*=0.0921). Table 3

**Table 3:** Number of patients developing toxicity composite endpoint according to age, performance, sex and previous radical pelvic radiotherapy

**Comparison groups**	**Number of patients reaching TCE**	***χ*^2^-test *P***
Age<70	101/267 (37.8%)	0.218
Age⩾70	33/72 (45.8%)	
Performance status 0–1	116/295 (39.3%)	0.793
Performance status 2–3	17/41 (41.5%)	
Male	89/201 (44.3%)	0.031
Female	45/138 (32.6%)	
Pelvic radiotherapy	23/51 (45.1%)	0.377
No pelvic radiotherapy	111/288 (38.5%)	

**Figure 1:**
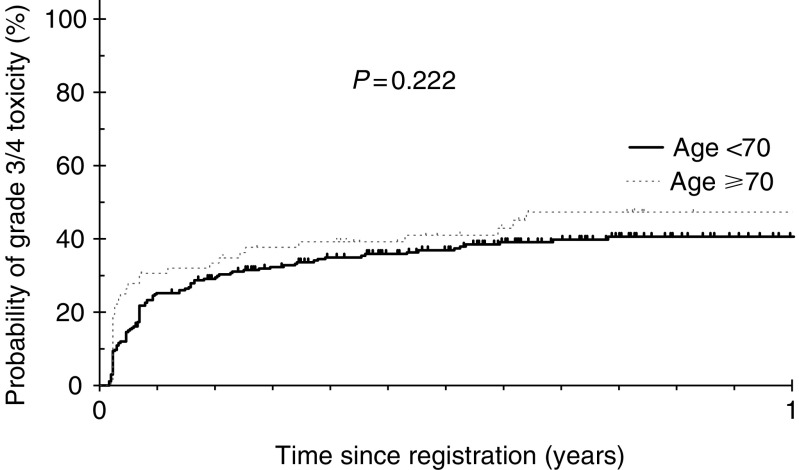
Time of occurrence of toxicity composite end point by age groups.

**Figure 2:**
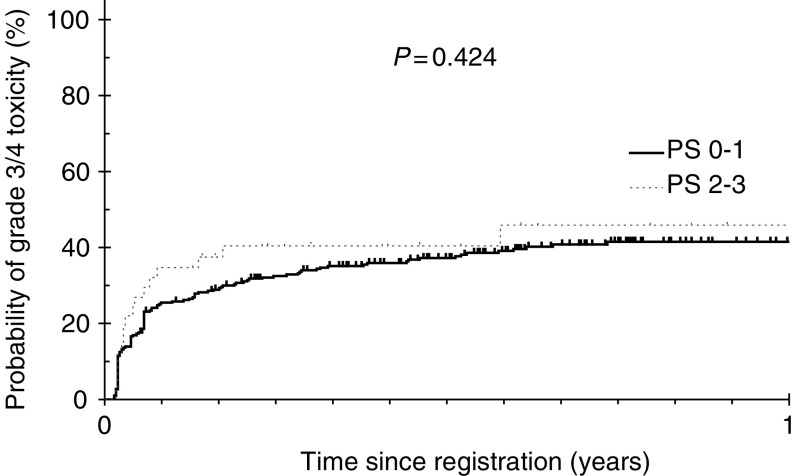
Time to occurrence of toxicity composite end point by performance status groups.

TCE: toxicity composite end point.

shows the number of patients developing TCE according to age, PS, gender and prior radical pelvic RT. There were no significant differences in the proportions of patients developing TCE by age, PS or prior RT (*P*>0.05). Time to occurrence of TCE was also similar by age (log rank *P*=0.222; Figure 1

) and PS (*P*=0.424; Figure 2

). Males experienced more toxicity than females (44.3 *vs* 32.6%; *P*=0.031), but this was not significant after controlling for other co-variates (*P*=0.06) in multivariate logistic regression modelling. Baseline bilirubin level was not significantly associated with TCE (*P*=0.149).

For the whole group, the objective response rate was 9.4% (95% confidence interval (CI): 6.3–12.6%). Table 4

**Table 4:** Objective responses for patients aged <70 compared to those aged ⩾70

	**<70 years old (*n*=267)**	**⩾70 years old (*n*=72)**	***P***
Complete response	0 (0%)	1 (1.4%)	
Partial response	24 (9%)	7 (9.7%)	
Stable disease	84 (31.5%)	28 (38.9%)	
Progressive disease	159 (59.6%)	36 (50%)	
Objective response rate (95% confidence interval)	9% (5.6–12.4%)	11.1% (4.9–20.7%)	0.585

shows the objective responses in patients aged <70 and ⩾70 years with no differences between the two age groups. For the whole group, the median survival was 9.1 months and 1-year survival was 35.3% (95% CI: 30.1–40.5%). There was no difference in survival between patients aged <70 years and ⩾70 years (log-rank test *P*=0.74; Figure 3

**Figure 3:**
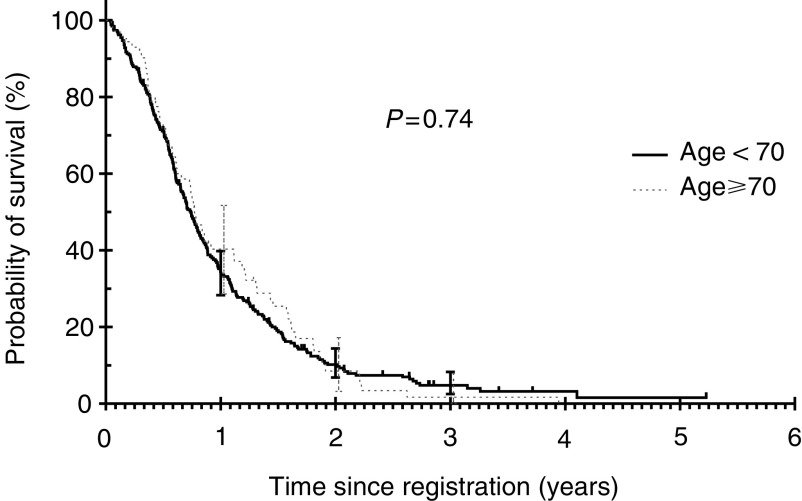
Overall survival by age groups.

). Table 5

**Table 5:** Multivariate analysis of prognostic factors on overall survival

**Factors**	**Hazard ratio**	**95% confidence interval**	***P***
>1 metastatic sites	1.275	1.01–1.61	0.041
Alkaline phosphatase >ULN	1.951	1.519–2.506	<0.001
Haemoglobin ⩽11g l^−1^	1.65	1.247–2.188	<0.001
White blood cell >11 × 10^9^ l^−1^	1.662	1.248–2.212	0.001
Previous pelvic radiotherapy	1.684	1.213–2.339	0.002
Performance status			0.092
Age^a^			0.734
Sex^a^			0.512

a Univariate analyses. ULN: upper limit of normal.

shows the multivariate survival analysis. Number of metastatic sites, serum alkaline phosphatase, haemoglobin, white blood cell count and prior pelvic RT were significant prognostic factors. Performance status (*P*=0.092) and gender (*P*=0.512) were not significant prognostic factors. There was no difference in survival between patients who developed the TCE and those who did not (*P*=0.317).

## DISCUSSION

Our study included 339 fluoropyrimidine and thymidylate synthase inhibitor-resistant CRC patients, all treated uniformly at 350 mg m^−2^ of irinotecan once every 3 weeks and this represents the largest single study with second-line irinotecan monotherapy reported to our knowledge. In this study we have shown that patients aged 70 or over had similar benefit and toxicity to irinotecan as younger patients. Poor PS and previous pelvic RT did not influence the incidence of irinotecan-related severe toxicity in these patients.

Although survival is the most important end point in evaluating new agents, insistence on its use as the only end point in clinical trials can result in the need for thousands of patients to be studied. Accordingly, composite end points have been increasingly used to increase the overall event rate and thereby reduce the number of patients needed to test specific hypotheses. The use of composite end points has resulted in widespread acceptance of therapies in heart failures and acute coronary syndrome (Cannon, 1997), although this approach is less utilised in oncological studies. To be used as part of composite end point, nonfatal end points should be clinically meaningful and related to an adverse outcome (Cannon, 1997). In our analysis, we were interested in evaluating irinotecan-related toxicity in predefined patient populations. The incidences of individual grade 3 & 4 toxicity were low (Table 2), despite our study being one of the largest conducted to date in this setting and this prevented us from comparing individual toxicities by specific patient groups. The gastrointestinal syndrome, comprised of diarrhoea, neutropenia, infection and nausea and vomiting, has been shown to be associated with early treatment-related or exacerbated deaths with irinotecan when used with bolus 5-FU/leucovorin (Rothenberg *et al*, 2001). These toxicities are well recognised as serious toxicities associated with irinotecan treatment and thus justified their use as components of our TCE.

The dose adjustment guidelines for irinotecan, produced by the manufacturer, recommend a reduced starting dose of 300 mg m^−2^ in patients aged ⩾70 years with PS 2. These guidelines have not, however, been incorporated into the Summaries of Product Characteristics. We have sought to use our independent data set to validate or refute these recommendations. Elderly patients are perceived to tolerate treatment more poorly and the benefits are less certain in elderly patients. In a systematic review of managing CRC in elderly patients, it was concluded that there is good evidence to support patients ⩽80 years of age having similar OS benefits with adjuvant 5-FU-based chemotherapy for colon cancer and with palliative first-line monotherapy for colorectal cancer, to younger patients (Au *et al*, 2003). Moreover, advancing age was not found to be related to the incidences of grade 3–4 nausea or vomiting, stomatitis or diarrhoea in patients treated with 5-FU-based adjuvant chemotherapy, although more leucopenia occurred with increasing age (Sargent *et al*, 2001). In the advanced disease setting, there was also no increase in toxicity in patients >70 years of age compared with younger patients (Popescu *et al*, 1999). However, the previously mentioned systematic review only included studies evaluating first-line palliative chemotherapy (Au *et al*, 2003). The effect of age in the second-line treatment of advanced CRC is much less evaluated. Our study showed that patients aged 70 or over had a similar survival and radiological response rate compared to younger patients without any increase in toxicity. However, the maximum age of patients recruited into our study was 80; therefore, our findings may not extend to octogenarians and nonagenarians.

In a pooled analysis of five phase II trials, 455 patients with metastatic CRC progressing on 5-FU were assessed for clinical efficacy and/or tolerance to irinotecan given at 350 mg m^−2^ every 3 weeks (Freyer *et al*, 2000). However, in three of these studies, treatment included an enkephalinase inhibitor against diarrhoea, racecadotril, which was assessed as the primary therapeutic intervention. In this pooled analysis, age was not significantly associated with disease response or stabilisation, although patients younger than 58 years old had worse progression-free survival compared to older patients. Overall survival was not assessed in this study (Freyer *et al*, 2000). Age was also not related to the occurrence of grade 3–4 neutropenia or diarrhoea (Freyer *et al*, 2000), consistent with our data. In a retrospective analysis of a randomised study evaluating a biweekly bolus irinotecan/5-FU/LV regimen, patients aged ⩾70 (*n*=17) did not suffer higher frequency of grades 3–4 toxicity compared to those aged under 70 (*n*=101) (Comella *et al*, 2003). Survival was also unaffected by age of patients. In another multicentre phase II study, it has been shown that chemotherapy with irinotecan or oxaliplatin-based treatment was feasible with manageable toxicity in the elderly (Aparicio *et al*, 2003). Similar data have also been found in first-line settings (Mitry *et al*, 2003; Rougier *et al*, 2003).

A meta-analysis of 2448 patients in five NCCTG clinical trials, using bolus schedules of 5-FU and LV, reported significantly more stomatits, diarrhoea, alopecia and leucopenia in women compared to men (Sloan *et al*, 2002). In addition, women experienced more toxicity than men consistently across all cycles of treatment and for all toxicities despite dose reduction after first cycles. These results suggested that women might be intrinsically more sensitive to 5-FU. However, this gender difference in toxicity is not limited to bolus 5-FU/LV schedules, but also extends to infused 5-FU (Tebbutt *et al*, 2003). In our analysis, we evaluated whether there were gender differences in efficacy and developing toxicity to irinotecan. Male sex was associated with a greater incidence of TCE, although this was not significant after controlling for other co-variates. No survival differences were seen between males and females. However, few other published data are available evaluating gender difference to irinotecan therapy (Innocenti *et al*, 2004). It is commonly accepted that abdomino-pelvic RT is associated with a greater incidence of irinotecan toxicities and many clinicians would elect to give a reduced starting dose. Previous RT has been shown to result in a greater incidence of grades 3–4 diarrhoea with irinotecan (*P*=0.046) (Freyer *et al*, 2000), although this observation was only of borderline significance. Our data and others did not support such a notion (Venook *et al*, 2003).

In our study, baseline serum bilirubin level did not influence the occurrence of TCE (*P*=0.149), although one has to note that our eligibility criteria would exclude patients with bilirubin level above 1.25 and 1.5 times the ULN in the absence and presence of liver metastasis respectively. A recent study has also found that baseline serum bilirubin did not reliably predict overall irinotecan-related toxicity in patients treated with irinotecan monotherapy within a phase III trial (Meyerhardt *et al*, 2004). Significant elevation of bilirubin is however associated with higher incidences of irinotecan-related toxicity (Raymond *et al*, 2002; Venook *et al*, 2003) and precludes normal starting dose of irinotecan to be used. Most recent research effort has focussed on UGT1A1 polymorphism as a determinant of irinotecan toxicity. Irinotecan is converted by carboxyl-esterase to its active metabolite, SN-38, which in turn undergoes glucuronidation by UDP-glucuronosyltransferase (UGT). UGT1A1 is the enzyme responsible for bilirubin glucuronidation of SN-38. UGT1A1 polymorphisms result in reduced UGT1A1 activity giving rise to genetic hyperbilirubinaemic syndromes such as Crigler–Najjar types I & II and Gilbert's syndrome and can lead to reduced gluronidation of SN-38. It has been found that patients either heterozygous or homozygous for UGT1A1^*^28, a variant sequence in the promoter region experienced more severe toxicity to irinotecan and had higher area under curve (AUC) SN-38 ratio compared to SN-38 glucuronide ratio (Ando *et al*, 2000, 2002; Iyer *et al*, 2002; Innocenti *et al*, 2004) Thus, interindividual differences in susceptibility to irinotecan toxicity can be partly explained by UGT1A1 mutation. However, whether starting with a reduced dose of irinotecan based on UGT1A1 polymorphism is an appropriate strategy requires prospective evaluation.

In our study, we have confirmed that the prognostic importances of some clinical and biological factors found in 5-FU based chemotherapy (Kohne *et al*, 2002) were also valid in irinotecan chemotherapy, that is, elevated alkaline phosphatase, low haemoglobin, elevated white blood cell count and >1 metastatic sites. Performance status 2 was not significantly associated with worse survival. We are unable to explain the reason why previous radical RT was a poor prognostic factor and this could be a chance finding that requires confirmation in an independent data set.

In conclusion, elderly and PS 2 patients derive the same benefit without experiencing more toxicity with second-line irinotecan treatment for advanced colorectal cancer. Pelvic RT did not result in additional toxicity. Our data do not support the recommendations to give a reduced starting dose to elderly and PS 2 patients.
